# Occupational Therapy Practice in Mainstream Schools: Results from an Online Survey in Switzerland

**DOI:** 10.1155/2019/3647397

**Published:** 2019-05-13

**Authors:** Vera C. Kaelin, Sylvie Ray-Kaeser, Stefania Moioli, Cornelia Kocher Stalder, Lietta Santinelli, Angelika Echsel, Christina Schulze

**Affiliations:** ^1^Rehabilitation Sciences, College of Applied Health Sciences, University of Illinois at Chicago, Chicago, IL 60612, USA; ^2^School of Health Professions, Institute of Occupational Therapy, Zurich University of Applied Sciences Switzerland, 8400 Winterthur, Switzerland; ^3^Department of Occupational Therapy, School of Social Work & Health Sciences, EESP, University of Applied Sciences and Arts of Western Switzerland, Lausanne 1010, Switzerland; ^4^Department of Business Education, Health and Social Care, University of Applied Sciences of Southern Switzerland, Manno 6928, Switzerland; ^5^Centro Ergoterapia Pediatrica CEP, Bellinzona 6500, Switzerland

## Abstract

The shift towards inclusive education in many European countries has led to structural changes that affect both schools and their related professionals aiming to support children's participation. While most European countries acknowledge inclusive education and its need, serious challenges exist to its implementation at a national and local community level. Interdisciplinary collaboration, including health and educational professionals, is seen as an imperative key principle for inclusive education services. To learn about the occupational therapist's contribution to inclusive education, the aim of this study was to describe the state of the art of occupational therapists' collaboration and services delivery in Swiss schools. Using an exploratory, cross-sectional study design, a web-based survey was sent to 509 occupational therapists in Switzerland resulting in 302 responses for data analysis using descriptive statistics. Findings show that nearly all participants (97%) collaborate with schools, and 49% of participants provided direct services within a mainstream school setting. These services were mainly funded by health insurance and focused on physical and social environmental adaptations. Despite reported collaboration between occupational therapists and schools, this study shows a need for changes in federal health and education legislation as well as innovative solutions for service delivery in schools.

## 1. Introduction

The United Nations Convention on the Rights of Persons with Disabilities [1] was signed in response to protect the rights of persons with disabilities and led to several changes within governmental regulations for many European countries. One major consequence of this commitment was a shift to inclusive education in regions where schooling systems were once characterized by separation. UNESCO [2] defines inclusive education as “schools accommodating all children regardless of their physical, intellectual, social, emotional, linguistic or other conditions” (p. 6). This definition is based on the understanding that restriction of full and effective participation in the society results from the interaction between the society such as attitudinal and environmental barriers and the person's ability [1, 2].

While most European countries acknowledge inclusive education and its need, serious challenges exist in achieving it at the national and local community levels [3, 4]. Therefore, the European Agency for Development in Special Needs Education [5] published key principles for practice to promote quality in inclusive education. Interdisciplinary collaboration is defined as integrating “knowledge and perspectives of different areas of professional expertise in order to consider issues holistically” ([6], p. 3) and is an imperative key principle for improving inclusive education services [5]. Interdisciplinary inclusive education teams may consist of teachers, special needs teachers, school-based speech therapists, and school-based occupational therapists as well as other professionals including the parents and child itself. For school-based occupational therapy, previous research has shown its value for inclusive education (e.g., [7, 8]).

The primary focus of school-based occupational therapy is to enable participation in school-based occupations such as writing during class, dressing for physical education, or playing during break times [9, 10]. Since participation in everyday occupations is considered as beneficial for children's development, health, and well-being [11, 12], occupational therapists have an important role in the school context. Best practice in school-based occupational therapy literature includes working collaboratively with school staff and parents to modify the context and the occupation and to provide occupational strategies to enhance performance and participation of children in school [13]. Internationally, the service delivery models and frameworks exist for use in school-based occupational therapy such as the “Response to Intervention” (RtI; [14]), the “Partnering for Change” (P4C; [7]), the “Occupational Therapy into Schools model” (OTiS; [15, 16]), and the “School-based Occupational Therapy Practice Framework” (SB-OT-PF; [17]).

Compared to more traditional models, where occupational therapists work one-to-one with a single child, these models and frameworks use a three-tiered approach of service delivery: tier 1 focuses on the whole school and/or class, tier 2 targets the group level, and tier 3 serves the individual child. An increased focus on the whole class and/or school allows to serve more children at the same time and may minimize the number of children in need of individual support [9] as well as limit waitlists for services [7]. On a class or school level (tier 1), occupational therapy services may involve supporting teachers to design alternative ways of performing school occupations if the traditional way is hindering children to reach the learning goals [7]. This way of working is in line with the educational initiative called Universal Design for Learning (UDL), which is aimed at enabling participation through the removal of physical and social environmental barriers [18, 19]. Occupational therapy services at a group level (tier 2) may involve differentiated instruction for a small group of children with challenges in occupational performance despite UDL interventions [7]. This, for example, can involve teaching strategies for cutting to a small group of children struggling with cutting. At an individual level (tier 3), a school-based occupational therapist works with a single child to support their occupational performance and participation in the natural environment and at the same time the children perform school occupations [7].

Compared to the United States of America, where school-based occupational therapy in inclusive school is well-implemented through federal legislation [20], European inclusive education and school-based occupational therapy is still an emerging practice (e.g., [17, 21]). This can be seen, for example, by the limited number of published articles on school-based occupational therapy practice in Europe. A search on current literature yielded only ten studies to date with the main focus on school-based occupational therapy practice in Europe (excluding studies for measurement validation). Out of these ten studies, five were conducted in England [15, 16, 22–24], two in Ireland [25, 26], two in Sweden [27, 28], and one in Portugal [29].

The two studies conducted in Sweden aimed at examining accommodation needs and participatory arrangements for students with physical disabilities in mainstream schools. Additionally, in the article by Hemmingsson et al. [28], cooperation of teachers and therapists concerning the arrangements for students and the organizational prerequisites for such cooperation was researched. In the study of Kalpogianni et al. [24], the focus was also on cooperation. They explored through a case study the joint working of occupational therapy and clinical psychology in a school setting. In the study of Dancza et al. [22], the focus was on the implementation of role emerging placements of English occupational therapy students in school settings. In the Portuguese study, Maia et al. [29] looked at interventions used by occupational therapists in schools to determine the most common intervention approaches and relevant aspects of the therapeutic process in schools. Three studies from England [15, 16, 23] and two from Ireland [25, 26] researched piloted occupational therapy interventions or services in mainstream schools. They involved the implantation of the Alert Program with 85 first year students in four Irish schools [25], the use of an innovative occupational therapy service (OTiS model) in two English schools [15, 16], and universal strategies to support motor development and functional skills in four English schools [23]. Patton et al. [26] looked at the collaborative application of a handwriting teaching method with 46 children with Down syndrome in Irish schools and the reality of collaborative practice with 44 primary teachers. Most of these studies highlight the importance of occupational therapist's collaborative engagement with other professionals within the school setting to support school participation of all children as well as inclusive education. This is in line with results from a recently conducted scoping review on recommended practices to organize and deliver school-based services for children with disabilities [30]. It synthesizes principles and implementation strategies including “multilevel and collaborative intervention service approaches that promote knowledge exchange and capacity building for everyone involved in the child's environment (parents, health professionals, teachers, and school staff) with training and the integration of well-coordinated partnerships between education, health services, and financial entities.” ([30], p. 11).

In Switzerland, pediatric occupational therapists mainly work in hospitals, clinics, and private practices and are not part of the mainstream school teams [31]. This is likely a result of the traditional role of occupational therapy in health care services [32]. There is a clear separation of the medical system and educational system in Switzerland, meaning that all services provided by schools are paid by the government and all medical services are funded by health care insurances. However, best practices in occupational therapy include working in the everyday life context of children, such as schools [9]. Lack of involvement in schools may limit occupational therapists' ability to impact participation in this context and collaboration with school staff. To our knowledge, it is not known how or to what extent occupational therapy practitioners in Switzerland collaborate with school staff and whether they provide school-based occupational therapy intervention congruent with inclusive philosophy.

Although referencing successful inclusive education and school-based occupational therapy interventions in different countries can be beneficial, each country must develop its own path to inclusive education [4]. The European Agency for Development in Special Needs Education [5], therefore, encourages systematic data collection on the national level to provide evidence and gain knowledge on the implementation and state of the art of inclusive education. Recent literature related to the current work of occupational therapists in Swiss schools could not be found. Knowledge about the state of the current occupational therapy practice with schools is necessary to understand the role of occupational therapists to support inclusive education in Switzerland. Therefore, the aim of this study was to describe and document the following:

1. The collaboration of occupational therapist with schools in Switzerland (frequency and way of contact, number of occupational therapists providing direct occupational therapy service in mainstream schools)
2. Structural and contextual aspects of occupational therapy services in mainstream schools in Switzerland (payment of service, the children supported)
3. Aspects of the occupational therapy process within mainstream schools in Switzerland (evaluation tools, setting, focus, and approaches for direct intervention)

## 2. Materials and Methods

This study used an exploratory, cross-sectional survey research design to examine pediatric occupational therapists' practices within Swiss mainstream schools. The Ethics Committee of Canton of Zurich confirmed no ethical concerns and therefore no need to submit a detailed ethical approval for this research project to the ethics committee in Switzerland.

### 2.1. Participants

Five hundred and three occupational therapists were initially recruited through the Swiss National Occupational Therapy Association (EVS) from the three main linguistic regions of Switzerland that were registered to work in pediatrics: 286 (57%) from the German-speaking, 117 (23%) from the French-speaking, and 100 (20%) from the Italian-speaking area. An email including the online survey in German, French, and Italian was sent to the identified pediatric occupational therapists. Because there is no official register with the existing number of occupational therapists working in pediatrics, participants were asked to forward the survey to their colleagues. Inclusion criteria for this study were as follows: occupational therapists working in a pediatric setting in Switzerland and reading and writing in at least one of the Swiss main languages (German, French, and Italian). By proceeding to complete the survey, participants were invited to confirm consent.

### 2.2. Instrument

The survey content was developed by the authors of this study who represent the three main linguistic regions of Switzerland. The questions were developed in English as a common research language and were critically revised by each author and a clinician with expertise in school-based occupational therapy. Professional interpreters were used to translate the survey into French, German, and Italian, and authors discussed and ensure correct and congruent terminology across the three languages, as well as reliability and validity of content. Each question was carefully scrutinized for accessibility (e.g., limiting length and complexity, avoidance of acronyms, abbreviations, and jargon that might not be familiar to all participants). The three-language versions of the survey were pilot-tested with a convenience sample to screen for clarity and potential online operating issues. Subsequent minor adjustments were made following participant feedback from the pilot test. The final self-administered survey contained 52 questions in total, consisting of 36 closed-ended questions in a multiple-choice format including prewritten answers and an option to provide additional open text if the respondent chooses “other” (e.g., “How are your school-based services financed?” with preselected answers: “health insurance,” “disability insurance,” “canton/community,” “special funds of schools,” “private payment of parents,” “partly not financed,” and “other”). Of those questions, 13 questions allowed only one answer and 23 allowed multiple answers. Four questions were “yes/no” questions and 12 were open-ended questions.

The survey started with five questions related to demographics and seven questions to occupational therapists' collaboration with schools (special schools, private or public mainstream schools) followed by two loops consisting of 16 identical questions: one loop for private/public mainstream schools and one loop for special schools. Participants answering “yes” to the following statements were directed to complete additional questions: “In the last year, I provided direct occupational therapy services in private/public mainstream schools” and “In the last year, I provided direct occupational therapy services in special schools.” Direct occupational therapy services were defined in the survey as being directly at school for any part of the occupational therapy process and for any client or client group. The additional questions addressed the context of schools, the assessments used, the interventions provided, the children supported, and the financial aspects of their services in private/public mainstream, respectively, special schools. The survey ended with eight final questions about further education in school-based occupational therapy, projects in any schools, and six general questions such as “I work in the school context because …”. Most of the questions were closed-ended, provided in a multiple-choice format allowing for either one or multiple answers. Six of the questions were open-ended and invited participants to provide additional information such as “These are challenges I encounter in my work in schools …”. Based on the research questions, this study focuses on the first part of the survey including demographics, questions about the collaboration of occupational therapists with schools, and additional questions about direct occupational therapy services provided private/public mainstream schools.

### 2.3. Procedures

Recipients received the link to the survey platform by an introductory email including information about the aim of the anonymous survey and a contact address for questions or feedback. The survey was available to potential participants between February 1^st^ and March 7^th^, 2016 (36 days). To maximize survey response rate, three weeks after the first email, recipients received a reminder to participate in the online survey if they have not done so yet.

### 2.4. Data Analysis

The questions of the survey used for this study were of closed-ended multiple-choice format. For analysis, quantitative data were downloaded into a Microsoft Excel (2016) spreadsheet and organized by data type and content. The closed-ended questions were analyzed using descriptive statistical analyses, more specifically by calculating percentages and data frequencies.

## 3. Results

In total, 309 occupational therapists completed the online self-administered survey, which is a response rate of about 61%. Because the actual number of pediatric occupational therapists in Switzerland is not known and the online survey was forwarded amongst colleagues, the percentage of response rate is approximate. Seven participants were excluded because they answered none or only questions about demographic data. Of the 302 remaining participants, some did not answer all questions; however, they were still included in our analysis. As a result, some questions were answered by more participants than others. Among the participants, 65% (*n* = 195) answered the survey in German, 21% (*n* = 64) in French, and 14% (*n* = 43) in Italian. Participants were mostly female (92%, *n* = 279) with the vast majority being self-employed (66%, *n* = 228) and a lower number being employed by an outpatient clinic (14%, *n* = 47), special school (8%, *n* = 27), and/or clinic or hospital (5%, *n* = 17) (Table 1). The question about occupational therapists work setting allowed selecting multiple answers to capture occupational therapists working in several settings. Participants' work experience ranges from 2 to 45 years, with a mean of 19.5 years.

### 3.1. Collaboration with Schools

Nearly all occupational therapists (97%, *n* = 292) reported collaborating with at least one school. Of those 292 participants, 35% (*n* = 101) were in contact with schools at least once a week. Thirty-eight percent (*n* = 112) of the occupational therapists reported to have been in contact with schools at least once a month but not weekly. Twenty-five percent (*n* = 73) had less than once a month contact with schools and the remaining 2% (*n* = 6) occupational therapists did not answer that question.

Occupational therapists reported using various ways to collaborate with schools. In a corresponding question allowing participants to select multiple answers, the majority reported having used telephone calls (22%, *n* = 265), emails (21%, *n* = 250), and/or participation at roundtable discussions (21%, *n* = 258) to collaborate with schools. Seventeen percent (*n* = 210) did class visits for observations at schools and/or visits for interventions (11%, *n* = 132). Some occupational therapists (3%, *n* = 39) gave presentations to school staff about occupational therapy and 3% (*n* = 32) also used other ways such as text messages. One percent (*n* = 16) of occupational therapists also stated that they have no contact with at least one school of the children they are working with.

Out of 302 occupational therapists, 145 (48%) responded to not only have collaborated with schools but also to have provided direct occupational therapy service in mainstream schools at least once in the past year. This means they have provided at least one part of the occupational therapy process directly at a mainstream school, either for an individual client or a group of clients.

Further results are based on data analysis conducted with the subsample of 145 occupational therapists that reported to have provided direct occupational therapy service in mainstream schools. If not stated differently, all the following results are based on questions that allowed participants to choose multiple answers.

### 3.2. Structural and Contextual Aspects of Occupational Therapy Services in Mainstream Schools

The 145 occupational therapists providing occupational therapy services directly within schools supported children having a variety of physical and cognitive impairments. The majority of children who were supported by occupational therapists were diagnosed with developmental coordination disorder (DCD, *n* = 115, 18%), attention deficit hyperactivity disorder (ADHD, *n* = 112, 18%), and autism spectrum disorder (ASD, *n* = 77, 12%). More information on diagnoses of children occupational therapists worked with at mainstream schools can be found in Table 2.

Participants stated that most of their occupational therapy services were paid either by health insurance (39%, *n* = 124) or by disability insurance (41%, *n* = 130). Fewer occupational therapy services were financed by the canton or community (3%, *n* = 11), by special funds from the school (1%, *n* = 4), by parents themselves (2%, *n* = 7), or were partly not covered (7%, *n* = 21) (Table 2).

### 3.3. Occupational Therapy Process within the Mainstream School

The participants were asked about different aspects of the occupational therapy process including how they evaluate children in mainstream schools, where their services were provided and whether their focus for intervention was mainly on the environment, the child, or the occupation.

The majority of occupational therapists used nonstandardized observation, interviews, and questionnaires during the occupational therapy process (see Table 2). Only 9% (*n* = 32) used standardized assessments such as the School Assessment of Motor and Process Skills (School AMPS; [33]), Movement Assessment Battery for Children (M-ABC; [34]), Test of Playfulness (ToP; [35]), Child Occupational Self Assessment (COSA; [36]), Evaluation of Social Interaction (ESI; [37]), Developmental Test of Visual Perception 2^nd^ Edition (DTVP-2; [38]), and Canadian Occupational Performance Measure (COPM; [39]) for intervention planning.

When providing occupational therapy services directly within schools, 49% (*n* = 124) of the occupational therapists chose the classroom, gym, or craft room, 15% (*n* = 38) chose the schoolyard during break time, and 8% (*n* = 20) chose other settings such as school kitchen, restroom, and/or school corridor. Twenty-eight percent (*n* = 71) in total mentioned to have worked with one child individually (24%, *n* = 60) and/or with a group of two children or more in separate rooms at mainstream school (4%, *n* = 11).

**Figure 1:**
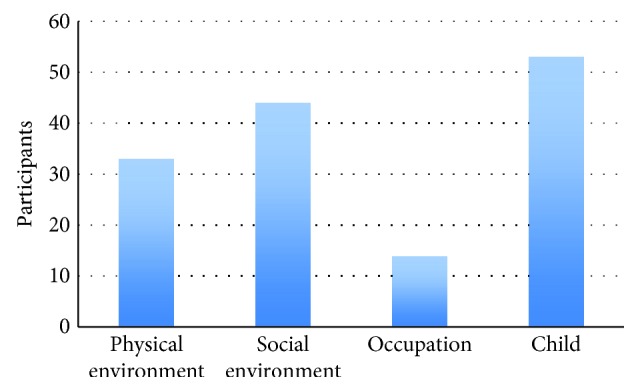
Main focus of the occupational therapy intervention. The bars represent the answers of participants (*n* = 144).

For a question allowing only one answer, more than half of the 144 responding occupational therapists (53%, *n* = 77) reported that their main focus of interventions was on the environment of the school setting. More specifically, 30% (*n* = 44) mentioned the social environment (e.g., consulting teachers or peers) as their main focus and 23% (*n* = 33) of the participants focused mainly on the physical environment (e.g., adapting the physical environment). Thirty-seven percent of the participants (*n* = 53) mainly focused on the competencies of an individual child (e.g., handwriting skills) and 10% (*n* = 14) on the adaptation of the occupation (Figure 1).

## 4. Discussion

The aim of this study was to gain knowledge regarding the current work efforts of occupational therapists in Swiss mainstream schools to understand occupational therapists' contributions to inclusive education. More specifically, this study describes and documents the collaboration of occupational therapists with schools in Switzerland and illustrates the structural and contextual aspects of their services in mainstream schools. Additionally, this study further investigates parts of the occupational therapy process provided in Swiss mainstream schools.

### 4.1. Collaboration with Schools

Because occupational therapists in Switzerland work mainly in medical settings such as hospitals, clinics, and private practices that are not necessarily connected to schools, collaboration with mainstream schools might be restricted. The results of this study show that despite their work settings which are mostly outside schools, the vast majority of occupational therapists taking part in this study collaborated regularly with schools. This collaboration mostly took place through phone, email, or by participating at roundtable discussions. Almost half of all participating occupational therapists provided occupational therapy services directly within mainstream schools at least once in the past year. The high number of occupational therapists collaborating with schools may be a result of the shift towards inclusive education in Switzerland, as a consequence of signing the UN Convention on the Rights of Persons with Disabilities in 2014 [1, 40]. Furthermore, the high number could be connected to a large number of literature supporting interdisciplinary collaboration and occupational therapy services in schools (e.g., [7]). Barriers to regular collaboration have been demonstrated such as receptivity to teaming, inflexible school schedules, or lack of time to meet and communicate [41]. For optimal collaboration, the use of multiple means to communicate, both in person and at distance, may be needed. However, the high number of occupational therapists in this study collaborating with schools is of significance when considering the importance of collaborative practice for inclusive education and for successful contribution of occupational therapy in schools (e.g., [5, 13, 41, 42]).

### 4.2. Structural and Contextual Aspects of Occupational Therapy Services in Mainstream Schools

Based on the results of this study, occupational therapists provided services to children diagnosed mainly with DCD, ADHD, or ASD. Interestingly, there were very few participants who stated to have provided occupational therapy services to children without a medical diagnosis. According to the service delivery models such as P4C [7] or RtI [14], in inclusive education, a medical diagnosis should not be a criterion whether a child is eligible to receive support or not. Having only a few occupational therapists in this study providing services to children without any medical diagnosis could be explained by the traditional method in Switzerland of referral to occupational therapists by a physician after diagnosis [43]. Insurance payment for occupational therapy services in Switzerland requires a physician's referral [44]. Accordingly, these structural aspects could contribute to the high number of occupational therapy services stated in this study that were paid by health or disability insurances.

### 4.3. Occupational Therapy Process within the Mainstream School

In this study, the evaluation tool occupational therapists used were mostly nonstandardized. This finding is of relevance because it shows that despite existing school-based occupational therapy assessments with evidence for its use in Switzerland [45], occupational therapists tend to use nonstandardized evaluation tools. An explanation for this result could be that occupational therapy services in mainstream schools are considered as an emerging field in Switzerland [46], and, therefore, occupational therapy assessments for use in schools might not be well known yet. This could indicate a need for knowledge translation efforts to make standardized school-based assessments with evidence for its use in Switzerland more known. Furthermore, it may show a need for the validation or development of additional school-based occupational therapy assessments in Switzerland. However, to find out about reasons for the use of nonstandardized and standardized assessment, further investigation is needed.

Of the occupational therapists that provided services directly within mainstream schools, almost two-third chose the classroom, the schoolyard, the gym or craft room, or other school contexts such as school kitchen, restrooms, and/or school corridors as their setting for their service. The other one-third reported having worked in a separate room at school, either with one child or with a group of children. Some authors argue for the necessity of pulling children out of their natural setting because interventions within their natural setting might have negative effects on students' self-esteem or could cause stigmatization [47]. Furthermore, a more easily controlled or less exposed setting such as a separate room is sometimes seen as needed for the mastery of skills necessary for a child's occupational engagement in school. However, when looking at it from a perspective of inclusion, support of children with special needs should optimally happen in their natural setting [1, 48]. This is in line with the current models of school-based occupational therapy practice, which recognize the need to move away from a pull-out, one-to-one, direct model of service delivery [49], towards more collaborative or consultative approaches [7, 8, 50] directly in the setting where problems occur [48]. The service delivery models such as RtI [14] or P4C [7] take this into account and promote occupational therapists' contribution at the classroom and school level by using Universal Design for Learning approaches. Using this model, occupational therapists can promote school participation and enable occupational engagement by modifying the environment or providing multiple means for learning. Good occupational therapy practice within the school suggested by literature includes collaborative teaming (i.e., coteaching), collaborative consulting, mentoring, coaching, and providing training or in-services to team members [13]. In Switzerland, where inclusive education is in its infancy, pulling children out of their natural setting might be chosen as a result of occupational therapists' habit of providing service in a separate setting or because of school's traditional habits of pulling children out of the classroom also for special needs education [51]. Alternatively, the relatively high number of occupational therapists stating to have seen children in separate rooms could be explained by structural aspects such as referral to occupational therapy service and its payment. In Switzerland, health and disability insurances are paid by individuals, which may lead to the notion that occupational therapy services are meant to be for a single child and their family only. Thus, occupational therapists could get caught in an ethical dilemma when providing occupational therapy services at the classroom or school level, when paid by a single child's health or disability insurances.

According to the code of ethics by the World Federation of Occupational Therapy [52], occupational therapists are required not only to comply with laws and regulations, such as of the funders of services, but also to provide services based on best evidence available. This leads to an interesting contradiction in countries such as Switzerland, where health and disability insurances are directed to one single child and his/her family, while evidence promotes service on a class or school level (e.g., [7, 14]). It may have led to the result of this study showing that occupational therapists (7%, *n* = 21) provided part of their services at mainstream schools on a voluntary basis. Providing unpaid services may have allowed them to work on a class or school level without violating regulations of funders. Additionally, this contradiction between regulations of health and disability insurances and existing evidence related to service delivery could be an explanation for the high number of occupational therapists in this study (53%, *n* = 77) choosing physical or social environmental adaptations as the main focus of their interventions at school. Adapting the environment for a child assigned to occupational therapy might make it more accessible for many children at the same time, as it is intended by the principles of Universal Design [1]. Therefore, it might have been another way for occupational therapists to comply with regulations of health and disability insurances and to work evidence-based at the same time. Alternatively, the result could be explained by existing evidence summarized by Barg et al. [53], highlighting the value of adapting the environment as an effective way to improve school participation of children.

Interestingly, only 10% (*n* = 14) of the participants answered their interventions focused on occupations, despite occupation as an important part of occupational therapy practice [54]. According to Coster et al. [55], components of occupation, such as physical, cognitive, and social demands of occupations, are the most frequent barriers to school participation. Not being part of the school team, occupational therapists might experience challenges maintaining this traditional focus of intervention. In Switzerland, teachers are usually in charge of selecting and carrying out school-related activities. In countries where school-based occupational therapy is established, enabling students to engage in school-related activities becomes a constant joint work of occupational therapists and teachers [48].

An alternative explanation for the small number of participants focusing mainly on occupation could be due to participants being unsure of the survey's distinction between adapting the environment and adapting the occupation or focusing on the child versus on the occupation. Depending on the literature, definitions and distinctions of occupation and environment vary (e.g., [56, 57]), which could have led to different interpretations by the participants when answering this question of the survey.

Limitations of this study include the rather high mean age and mean years of work experience of the study participants, which represents a slightly older group of occupational therapists in Switzerland, which could be a bias to the results. Similarly, most of the participants worked in a private practice, which might not be representative of occupational therapists in Switzerland. Another limitation of the study is related to data analysis. For questions about the collaboration of occupational therapists with schools, the data analysis was done with the whole study sample. Because the other questions were related to direct occupational therapy service within mainstream schools, data analysis for those questions was based on a subsample of 145 participants only. Demographics of the whole sample and the subsample may differ.

However, the survey gives a good overview of occupational therapy services in Swiss schools and whether their services are in line with the United Nations Convention on the Rights of Persons with Disabilities [1]. Overall, the results show strong compliance of occupational therapy services with the United Nations [1]; however, regulations such as payment through health and disability insurances can interfere with occupational therapists application of evidence-based practice such as services on class or school level including Universal Design approaches. To support occupational therapists to contribute effectively to inclusive education in Switzerland, as it is a good practice (e.g., [7, 8]) and evident in other countries (e.g., [48]), structural aspects need to be reflected and the innovative service organization models need to be found and followed by research.

## 5. Conclusions

Although occupational therapists are not typically part of the school system in Switzerland, many find ways to support inclusion through collaboration and service provision in schools. Their services mainly focus on the school environment rather than the child's disability, which is congruent with inclusive philosophy. However, occupational therapy services were mainly provided to children with medical diagnoses and only a few occupational therapists stated to have supported children without a medical diagnosis. This could lead to the assumption that occupational therapy services within schools are mostly provided on an individual level and less on a group or class, respectively, school level. The results of this study show not only the promising first steps towards occupational therapy's support of inclusive education in Swiss schools but also a need for changes in federal health and education legislation and innovative solutions to service delivery that consider challenges to collaborative practice.

## Figures and Tables

**Table 1:** Demographic characteristics of the participants.

Demographic characteristics	*n* (%)
Gender	
Female	279 (92)
Male	16 (5)
Missing value	7 (2)

Survey language	
German	195 (65)
French	64 (21)
Italian	43 (14)

Work setting (multiple answers)	
Self-employed	228 (66)
Employed outpatient clinic	47 (14)
Employed (clinic hospital)	17 (5)
Employed (special school)	27 (8)
Others	27 (8)

**Table 2:** Structural and contextual aspects of occupational therapy services in mainstream schools.

Structural and contextual aspects	*n* (%)
Most common diagnostic groups (multiple answers)	
Developmental coordination disorder (DCD)	115 (18)
Attention deficit hyperactivity disorder (ADHD)	112 (18)
Autism spectrum disorder (ASD)	77 (12)
Cerebral palsy (CP)	74 (12)
Developmental delay	73 (12)
Learning disability	70 (11)
Down syndrome	19 (3)
Traumatic brain injury (TBI)/oncology	12 (2)
Multiple disabilities	25 (4)
Not diagnosed difficulties	29 (5)
Others not specified	27 (4)

Finances (multiple answers)	
Health insurance	124 (39)
Disability insurance	130 (41)
Canton/community	11 (3)
Special fund of schools	4 (1)
Private payment of parents	7 (2)
Partly not financed	21 (7)
Other	5 (2)
Value missing	19 (6)

Assessments (multiple answers)	
Observations	142 (38)
Interviews	119 (32)
Questionnaires	59 (16)
Films	21 (6)
Standardized assessments	32 (9)

## Data Availability

The nature of the data is an Excel file. To access the data, please contact the authors of the article.
